# Neural Energy Supply-Consumption Properties Based on Hodgkin-Huxley Model

**DOI:** 10.1155/2017/6207141

**Published:** 2017-02-16

**Authors:** Yihong Wang, Rubin Wang, Xuying Xu

**Affiliations:** Institute for Cognitive Neurodynamics, East China University of Science and Technology, 130 Meilong Road, Shanghai, China

## Abstract

Electrical activity is the foundation of the neural system. Coding theories that describe neural electrical activity by the roles of action potential timing or frequency have been thoroughly studied. However, an alternative method to study coding questions is the energy method, which is more global and economical. In this study, we clearly defined and calculated neural energy supply and consumption based on the Hodgkin-Huxley model, during firing action potentials and subthreshold activities using ion-counting and power-integral model. Furthermore, we analyzed energy properties of each ion channel and found that, under the two circumstances, power synchronization of ion channels and energy utilization ratio have significant differences. This is particularly true of the energy utilization ratio, which can rise to above 100% during subthreshold activity, revealing an overdraft property of energy use. These findings demonstrate the distinct status of the energy properties during neuronal firings and subthreshold activities. Meanwhile, after introducing a synapse energy model, this research can be generalized to energy calculation of a neural network. This is potentially important for understanding the relationship between dynamical network activities and cognitive behaviors.

## 1. Introduction

Coding and decoding of neural information have been the core problem in cognitive neural science [1–6]. Phase coding, frequency coding, and group coding have been established to deal with this problem [1]. These issues have been of interest to scientists from around the world. Unfortunately, these techniques are limited in scope and are difficult to complete successfully [7, 8]. Currently, there is no complete theory for neural coding and decoding to direct the research of global brain activities. The reason is that these local coding theories do not include the cross influence of large-scale neural activities. The neurodynamics are nonlinear, which makes it very hard to perfectly analyze the neural coding and decoding problem [8–18]. Neural activities and neural information processes should follow the principles of energy minimization and information transmission efficiency maximization [19]; in other words, neural system should be restricted by energy minimization regardless of suprathreshold or subthreshold activity. This is the economical essence of neural system because of evolution. Information transmission efficiency must maximize the energy utilization in a neural system; this property is determined by the high efficiency of neural system [19]. However, it is difficult to describe on a quantitative basis neural metabolic energy, neural electric energy, and the relationship between them. Some research has calculated neural energy, but these descriptions are restricted on the electrochemistry level [20, 21]. Thus, neural energy cannot be coupled to neural coding at the network level. This research has helped us to understand the neural energy consumption and transformation, but they are not related to information coding by neuron group activity.

Researchers have proposed a new method to study neural coding through the use of energy levels to compensate for the problems mentioned above [22]. A biophysical model has been constructed to describe the relationship between bioenergy of the brain and the neural information processes of the prefrontal cortex. Furthermore, quantitative connections between firing patterns and neural energy evolutionary process have been found. Based on these unique connections, researchers have proposed the concept of energy coding [19, 23] and calculated the energy of a single neuron [24]. Some interesting discoveries have been found during the study of the energy distribution properties of structural neural networks [24]: (1) the theory of neural energy coding is based on the use of a global concept of energy; (2) neurons release their stored energy within a very short time (negative energy) at the beginning of firing action potential, after which the oxyhemoglobin provides them with biological energy, and this mechanism contradicts the traditional theory of pure energy consumption in neurons; (3) the distribution of the negative energy, as assessed by parameter studies, reflects the neural network parameters and neural oscillation with a high consistency. These ideas have laid the foundation for energy coding research of the functional neural network.

Scientists studying neural energy consumption [6, 20, 25–27] have analyzed the demand of adenosine triphosphate (ATP) in the coding of certain bits of information from experiments and computational model and provided the efficiency relationship between information transformation and energy metabolism in certain conditions. However, these remarkable works focused only on one single prospective of energy consumption of a neuron, or the energy support during neural information process. They ignored the other equally important reversal problem, which is, from energy metabolism and utilization properties, whether we can decode some information of stimulus and neural response. In this research, we analyze this problem by energy coding method. The most important neural activity is the changing of membrane potential of a neuron, which depends on the work of ion pumps. Among these pumps, the Na^+^/K^+^ pump is the most crucial one and it consumes most of the metabolic energy. Thus, energy consumption by the Na^+^/K^+^ pump reflects the metabolic energy used by neurons at the same time. This energy transforms to potential energy to maintain membrane potential and is consumed by electrical activity. Finally, the energy dissipates and turns into heat. Thus, energy is supplied to the ion pump by ATP; then the ion pump works to transport ions against the concentration gradient to preserve electrical potential energy. This process is equivalent to charging a battery. When an action potential occurs, ions move along the electric potential difference, and the potential energy preserved in the membrane is released and turned into joule heat due to the resistance effect of ion channels. In general, energy is supplied by ATP and consumed by the ion channel. Apparently, energy should be conserved during lager scale of time, but in small time interval energy supply and consumption are not really matched every moment. This property makes it possible to study brain activity status based on energy supply.

## 2. Model and Method

From the former discussion, it can be deducted that energy supplied to a neuron could be measured by the amount of ATP consumed by the ion pump, which is the direct energy source of a neuron. The energy consumed by a neuron can be measured by the joule heat transformed from electric potential energy, so that we can calculate this energy consumption from membrane potential, Nernst potential, and ion current. Most metabolic energy released by ATP is provided to the Na^+^/K^+^ pump, so energy consumed by the Na^+^/K^+^ pump could represent the energy supply to a neuron. On the one hand, for every 3 Na^+^ ions pumped out of a cell membrane, one ATP molecule is consumed [26]; each mole of ATP molecules can release between 46 and 62 kJ free energy [28]. After Na^+^ flow into neuron during neural activity, the Na^+^/K^+^ pump will expel at least the same amount of Na^+^ to reset the resting membrane potential. Thus, as long as we calculate the amount of Na^+^ flow into neuron, we can estimate the ATP consumption [29] and the final energy supply to a neuron. On the other hand, based on a reasonable neuron ion channel model, joule heat generated by electric activity of a neuron can be obtained by the integral of electric power of each ion channel, which is the product of the electric potential and the current [20]. This integral represents the energy consumption of a neuron. Fortunately, all these characters can be obtained by Hodgkin-Huxley model (H-H model).

**Figure 1:**
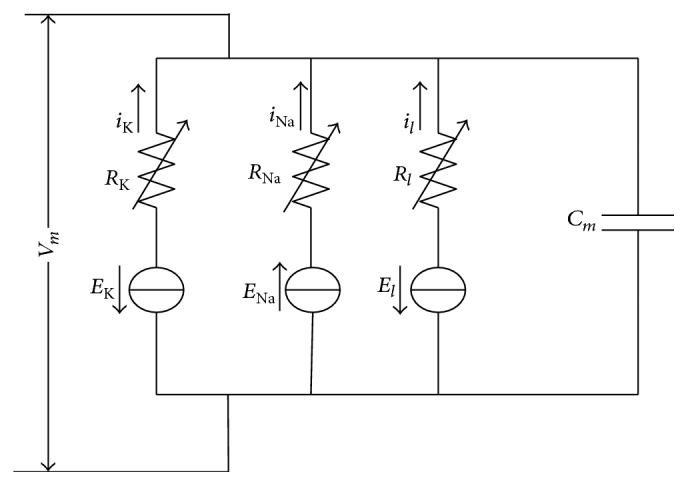
Circuit of H-H model.

Circuit of H-H model is depicted as in Figure 1.

The differential equation is

(1) CmdVmdt=glEl−Vm+gNam3hENa−Vm+gKn4EK−Vm+I,

where *C*_*m*_ is membrane capacitance of a neuron, *V*_*m*_ is membrane potential, *E*_Na_ and *E*_K_ are Nernst potentials of Na+ and K+, and *E*_*l*_ is the potential while leakage current is zero. *g*_*l*_,  *g*_Na_, and *g*_K_ are, respectively, leakage conductance, Na+ channel conductance, and K+ channel conductance. These three kinds of conductance are described as the following nonlinear differential equations:

(2) dndt=αn1−n−βnn,dmdt=αm1−m−βmm,dhdt=αh1−h−βhh,

where

(3) αn=0.0110+Vm−Vrexp⁡10+Vm−Vr/10−1,βn=0.125exp⁡Vm−Vr80,αm=0.125+Vm−Vrexp⁡25+Vm−Vr/10−1,βm=4exp⁡Vm−Vr18,αh=0.07exp⁡Vm−Vr20,βh=1exp⁡30+Vm−Vr/10+1.

And *V*_*r*_ is the resting membrane potential.

The action potential firing process is as follows: (1) After postsynaptic neuron receiving impulse from presynaptic neuron, the neuron membrane increases its permeability to Na^+^ changes and begins to depolarize (subthreshold activity); (2) permeability further increases, with enormous Na^+^ flow inward, and membrane potential rises rapidly (suprathreshold activity); (3) permeability to Na^+^ decreases and permeability to K^+^ increases, and repolarization begins; (4) permeability to K^+^ continues to increase and K^+^ flows outward until hyperpolarization; (5) after permeability to K^+^ decreases, membrane potential rises to a resting level.

As a result, energy supplied by ATP during action potential or subthreshold activity can be calculated based on the H-H model:

(4) $$\begin{array}{c} E_{s}=\frac{\lambda }{eN_{A}}\int_{t}g_{Na}m^{3}h\left(\;E_{Na}-V_{m}\;\right)dt, \end{array}$$

where *λ* is amount of energy released by one-mole ATP, *e* is the elementary charge, which is 1.6 × 10^−19^ coulombs, and *N*_*A*_ is Avogadro constant, and the integrand is the current of the Na^+^ channel.

Energy consumption by a neuron based on H-H model can be determined by the following method [20].

At a particular moment, electric energy contained by a neuron is accumulated in membrane capacitor and equivalent batteries generated by Nernst potentials of ions, which is

(5) $$\begin{array}{c} E\left(\;t\;\right)=\frac{1}{2}C_{m}V_{m}^{2}+H_{Na}+H_{K}+H_{l}, \end{array}$$

where *C*_*m*_ and *V*_*m*_ are membrane capacity and potential and the last three terms are energy accumulated in batteries which are difficult to calculate directly. Thus, we focus on electrical power which is the time rate of change of total energy:

(6) $$\begin{array}{c} \frac{dE}{dt}=C_{m}V_{m}\frac{dV}{dt}+i_{Na}E_{Na}+i_{K}E_{K}+i_{l}E_{l}, \end{array}$$

where the last three terms are ion currents and Nernst potentials, respectively. After applying (1) we obtain the following:

(7) dEdt=VmI+iNaENa−Vm+iKEK−Vm+ilEl−Vm.

Integrating this equation at a particular time interval, we are able to calculate the energy consumed by a neuron during this time-period:

(8) Ec=∫tVmI+iNaENa−Vm+iKEK−Vm+ilEl−Vmdt.

Apparently, *E*_*c*_ = *E*(*t*) + *C*, where *C* is an unknown constant determined by the electrophysiological features of neuron. This means we can calculate energy consumption of a neuron during a time interval (especially during electric activity), although total energy contained in neuron is unknown to us.

As soon as energy supply and consumption are calculated, energy efficiency can be defined by percentage of energy consumption over supply:

(9) $$\begin{array}{c} \eta =\frac{E_{c}}{E_{s}}\times 100\%. \end{array}$$

Energy efficiency may differ a lot at different neural activity status, so it can be applied to reflect activity state of a neuron and even neural networks. In our work, we found that this parameter can serve to distinguish supra- and subthreshold activity.

Besides, by calculation it is easy to find that Na^+^ and K^+^ currents consume most of electric potential energy. Thus, it is valuable to analyze energy usage detail of these two ion currents, energy consumption relationship between them, and its evolution over time. This will help us grasp the details of energy transformation in a neuron. We will analyze power ratio of these two ion currents (Na^+^ power over K^+^ power) and the change over time of power percentage over total power (ion power over total power). Furthermore, the inner product of Hilbert space can be applied to define the synchronicity of their energy consumptions to analyze their spatiotemporal relationship. Synchronicity of energy consumption *τ*(*E*) is

(10) τE=∫tiNaENa−Vm×iKEK−Vmdt∫tiNaENa−Vm2dt×∫tiKEK−Vm2dt.

And Ψ(*E*) = arccos⁡(*τ*(*E*)) is the phase difference of energy consumption. Similarly, ion currents synchronicity *τ*(*I*) and phase difference Ψ(*I*) are

(11) τI=∫tiNa×iK dt∫tiNa2 dt×∫tiK2 dt,ΨI=arccos⁡τI.

Besides the energy contributed to Na^+^/K^+^ pump by ATP hydrolysis, the external stimulus current also provides some energy. This amount of energy is so small that it can be ignored during the calculation of energy supply. However, this stimulus energy is like a blasting fuse. If it leads to a spike or action potential, this energy change can be ignored. If the neuron merely activates subthreshold, it will be worthwhile to consider how much this energy contributes to subthreshold activity. As a result, we can calculate the ratio of stimulus energy over total energy consumption to detect whether this energy can be ignored and determine if this ratio can be distinguished from supra- and subthreshold activity.

## 3. Results

According to the described method, we use MATLAB (R2013a) to perform numerical simulation. Parameters in H-H model are taken the typical value: maximum Na^+^ conductance *g*_Na_ = 120 mS/cm^2^, maximum K^+^ conductance *g*_K_ = 36 mS/cm^2^, leakage conductance *g*_*l*_ = 0.3 mS/cm^2^, and Nernst potentials are 50 mV, −80 mV, and −56 mV, respectively. Resting membrane potential is 67.3 mV.

### 3.1. Ion Currents and Energy during Firing

Initially, we set the stimulus current at *I* = 3 *μ*A/cm^2^ and the last at 5 ms. A typical action potential is generated, which is represented in Figure 1.

During the action potential, the ion currents that occurred in each of the ion channels are shown in Figure 3. If the outward direction is positive, Na^+^ current should be negative. However, in Figure 3 it is depicted in absolute value (reversed) to be compared more easily. The lines with color of red, black, yellow, and fuchsia represent Na^+^, K^+^, leakage, and stimulus currents, respectively. As shown in Figure 3, Na^+^ and K^+^ currents are much stronger than leakage and stimulus currents; thus it is reasonable to focus on the ion currents and energy consumption of these two ion channels. Meanwhile, the waveforms of these two currents result in the cross-membrane charge being largely neutralized. The synchronicity of ion currents defined previously is *τ*(*I*) = −0.987, and negative value means that the current directions are opposite, overall. Phase difference Ψ(*I*) = 170.7°, which means the phases of these currents are almost completely opposite. The net current is depicted in Figure 4. In addition, we set the outward direction as positive. After about 4.5 ms, the net current has an inward spike, because Na^+^ burst into the membrane and K^+^ conductance across the membrane have not risen. The integral of this net current (area between the curve and time axis) shows that the net electric charge across the membrane during this action potential is 15 nC/cm^2^. In other words, 15 nC of positive charge transfers out of the membrane per cm^2^. Interestingly, the stimulus current injected into neuron is 3 *μ*A/cm^2^ and lasted 5 ms, and 3 *μ*A/cm^2^ times 5 ms equals 15 nC/cm^2^, which means, by stimulus current, same amount of electric charge was injected into cell and the cell stayed in electric neutrality. This suggests the following fact: all the ion migration across the membrane during the whole phases of action potential serves to neutralize the external stimulus charge injected into the cell.

**Figure 2:**
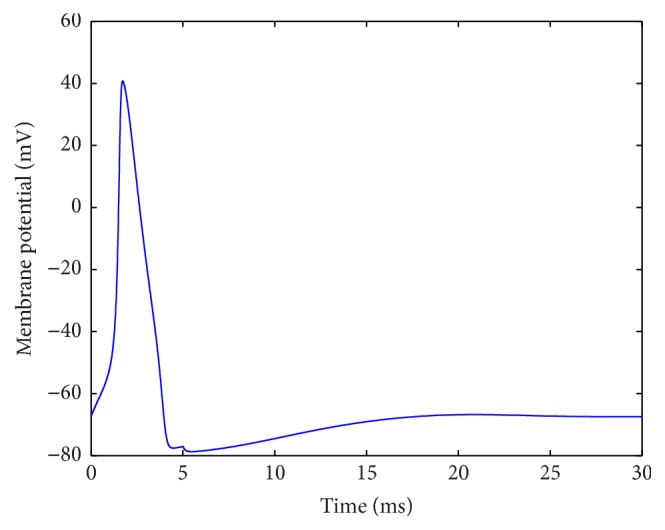
A typical action potential.

Energy consumption of each ion channel during action potential is one of the most important problems.  Figure 5 shows the electric power of each ion channel during this process. Green line is the total power and red, black, yellow, and fuchsia lines are powers of Na^+^, K^+^, leakage, and stimulus currents, respectively. It is evident that most of the energy is consumed by Na^+^ and K^+^ channel. Meanwhile, compared with Figure 2 it is apparent that the waveforms of Na^+^ and K^+^ are quite different from current waveforms. Currents climb to the peak almost at the same time, and peak values are barely different. Meanwhile, the power peak of Na^+^ clearly lags behind K^+^, and the value is lower. The synchronicity of power *τ*(*E*) is 0.782, and the phase difference Ψ(*E*) is 38.5°. Peak value of Na^+^ power is 66% of that of K^+^. While comparing Figures 2 and 5, we can determine that the peak membrane potential and the peak total power do not appear simultaneously. Power has little lag and the peak of total power occurs between the peaks of Na^+^ and K^+^ power. Leakage conductance and Nernst potential are set to be constants, so the trend of leakage current and power is similar with membrane potential.  Figure 6 shows the power ratio of N^+^ and K^+^ (Na^+^/K^+^) at each moment to reveal their relationship in detail. The curve above the blue horizontal line stands for the moments that Na^+^ power exceeds K^+^ power. Although peak value of K^+^ power is larger than Na^+^, it has a sharper wave, so, before resting potential restored, Na^+^ power is larger than K^+^ in majority of time and the ratio curve crosses the equality line several times which means power ratio fluctuates a great deal. This is a significant difference from the subthreshold activity.

Figure 7 depicts ratios of these two channel powers over total power at each moment. When the membrane potential reaches its peak and is restored to the resting level, the Na^+^ channel occupied almost all the power consumption for two times and showed a bimodal distribution. In contrast, the K^+^ channel maintained the highest power only once when the membrane potential has risen to the highest level. This shows that when membrane potential is at the highest, all the energy is consumed by K^+^ channel.  Figure 8 shows the sum of these two ratios in Figure 7. During action potential (before 7.5 ms), Na^+^ and K^+^ channels consumed almost all the electric power supply. After membrane potential is restored to resting level, total power is too low to be considered. Thus, Na^+^ and K^+^ channels indeed consume almost all the electric energy during neuron firing as we guessed. As a result, these energy consumption properties of the two channels are a precise reflection of the neuron activity in detail.

**Figure 3:**
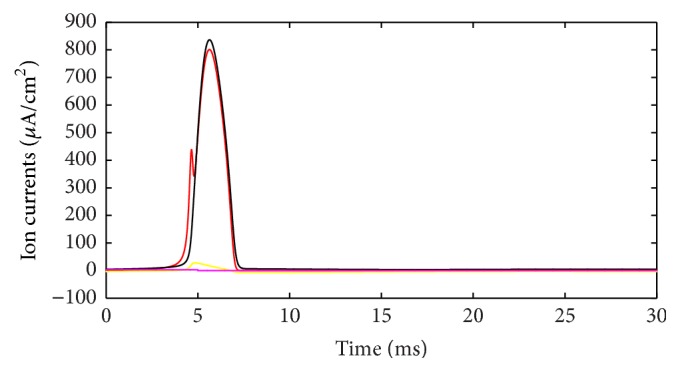
Different ion currents during action potential.

The current strength and power of external stimulus are negligible in comparison with others. This can be observed visually in Figures 3 and 5. The calculation suggests that the peak of ratio of total power over stimulus power can reach hundreds of thousands (Figure 9). This is a typical “blasting fuse” effect of stimulus current. Due to the huge power difference, stimulus energy can be ignored when we consider the energy supply to a neuron. Meanwhile, net ion current across the cell membrane can only neutralize electrical charge injected by a stimulus current. Such a large difference occurs during analyzing relationship between neural activity and stimulus current in perspectives of electric charge and electric power. This is quite an interesting phenomenon.

By ion-counting method, which integrates Na^+^ current, we can get that 1.429 *μ*C (per cm^2^) of positive electric charge flows into cell membrane in the form of Na^+^ during an action potential. These are 8.918 × 10^12^ of Na^+^ ions. Na^+^/K^+^ pumps have to pump all these influx Na^+^ out of membrane again to maintain resting potential and electric neutrality, so one-third of number of Na^+^ is the number of ATP molecules consumed. Then, the number of moles of ATP is 4.94 × 10^−12^ mol. In final, the energy supply to a neuron by ATP during one action potential is 2.468 × 10^−7^ J (every mole of ATP releases 50 kJ free energy). Meanwhile, integrating the total power with respect to time (the area below green line in Figure 5), we obtain the consumed electric power, which is 1.879 × 10^−7^ J in this case. Thus, it can be concluded that, during action potential, electric power consumed by ion channels is approximately 76% of the energy supplied by ATP at the same time. This is the energy efficiency of a neuron during suprathreshold activity.

### 3.2. Ion Currents and Energy during Subthreshold Activity

**Figure 4:**
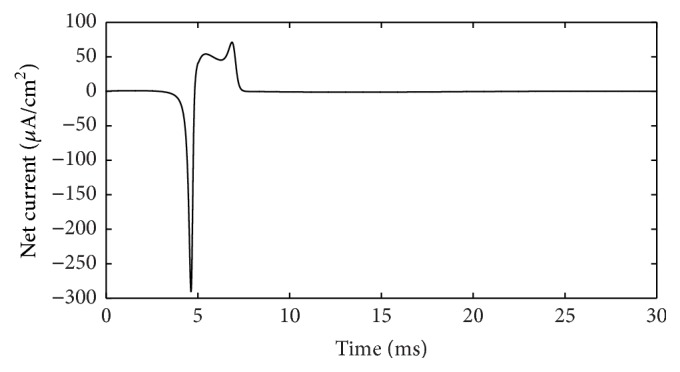
Net current (suprathreshold).

In this section, we will examine the subthreshold current and energy properties of a neuron when stimulus is weak and no action potential was fired. As stimulus current strength is 2.5 *μ*A/cm^2^ and lasts 3 ms, the membrane potential is shown in Figure 10, which rises to 5 mV first and then drops to a resting level. Similar to Figure 3, Na^+^ current is shown as an absolute value in Figure 11 for comparison. The meaning of colored lines is the same as before. The strength of the two ion currents is almost the same order of magnitude as stimulus and leakage currents, which is clearly different from the firing state (Figure 3). Another difference is Na^+^ current is weaker than K^+^ for the entire time-period for the comparison of Figure 3, in which the two curves cross each other several times. However, their waveforms overlap. The synchronicity is −0.90 and phase difference is 154.16°, meaning that the antiphase is deduced a little. Net current is depicted in Figure 12, the curve is smoother, and the negative peak disappeared compared with Figure 4. This is because Na^+^ channel is not fully open and Na^+^ current is not strong enough. Similarity, the peak occurs after 5 ms, which is the moment that Na^+^ and K^+^ current have decayed. The integral of this net current shows the net positive electric charge crossing out of the membrane during this action potential is 7.52 nC/cm^2^. Notice that 2.5 *μ*A/cm^2^ of stimulus current lasts 3 ms and 7.5 nC/cm^2^ positive charge is injected into the cell membrane. They are still the same, basically. This suggests that during subthreshold activity the cell maintains electric neutrality at suprathreshold. The ion migration across the membrane during subthreshold activity also serves to neutralize the external stimulus charge injected into the cell.

**Figure 5:**
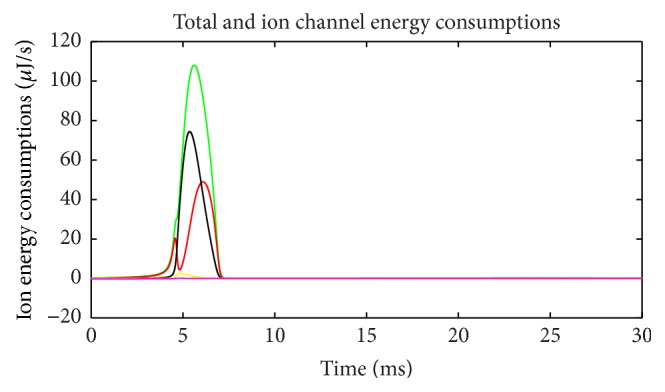
Energy consumption of each ion channel (suprathreshold).

**Figure 6:**
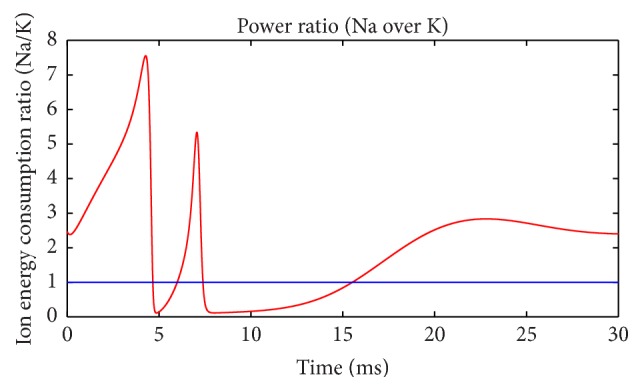
Power ratio of Na^+^ and K^+^ channels (suprathreshold).

**Figure 7:**
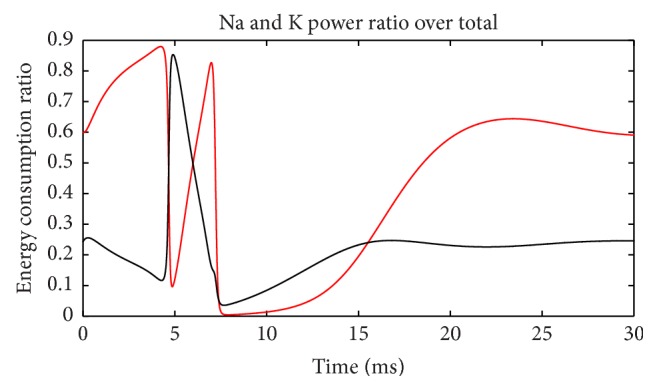
Power ratios of Na^+^ and K^+^ over total (suprathreshold).

**Figure 8:**
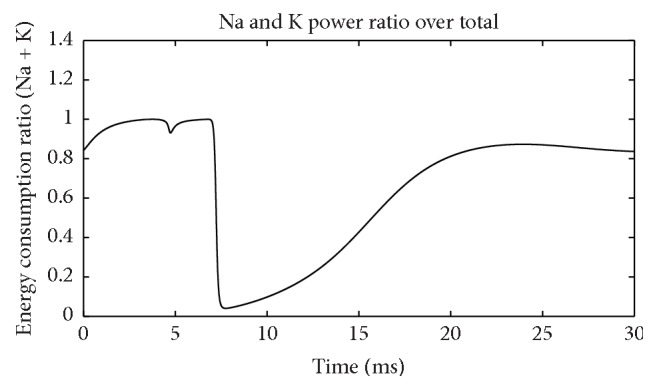
Sum of Na^+^ and K^+^ power over total power (suprathreshold).

**Figure 9:**
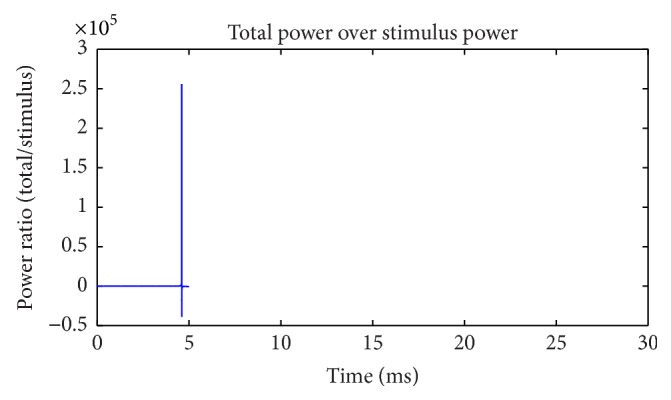
Ratio of total power and stimulus power (suprathreshold).

**Figure 10:**
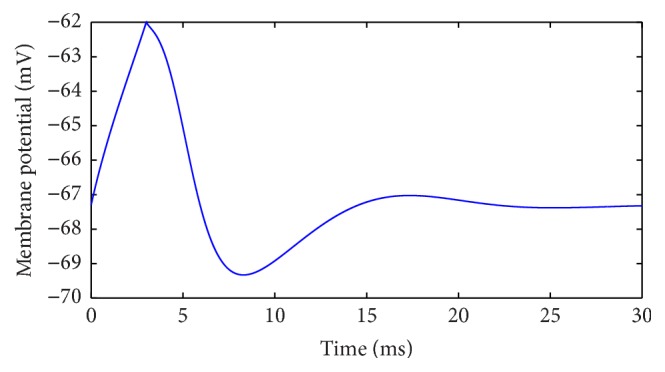
Subthreshold membrane potential.

**Figure 11:**
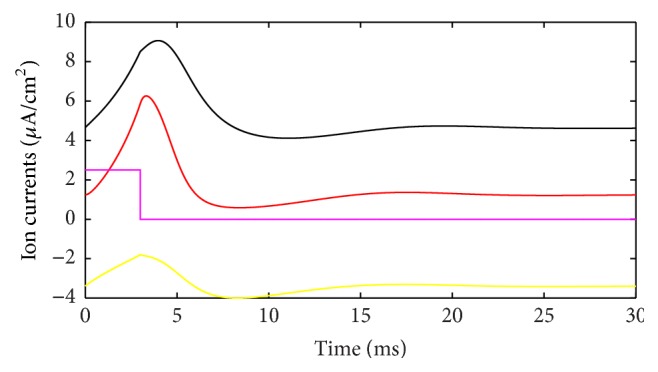
Subthreshold currents of different ions.

**Figure 12:**
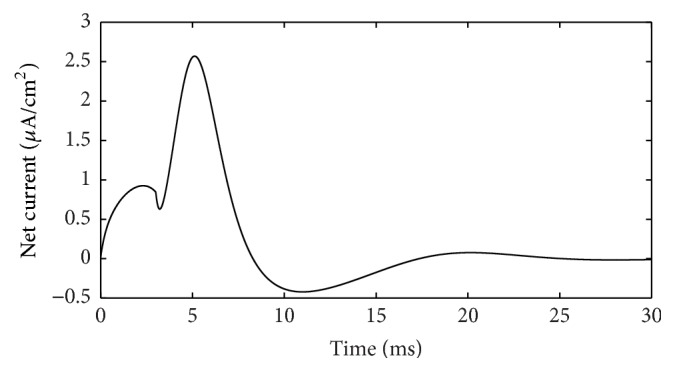
Subthreshold net current.

**Figure 13:**
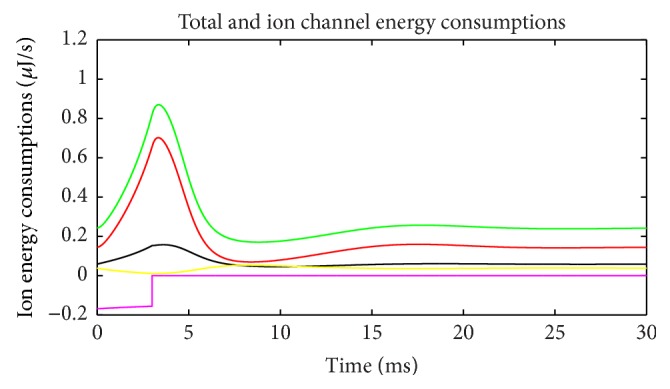
Energy consumption of each ion channel (subthreshold).

**Figure 14:**
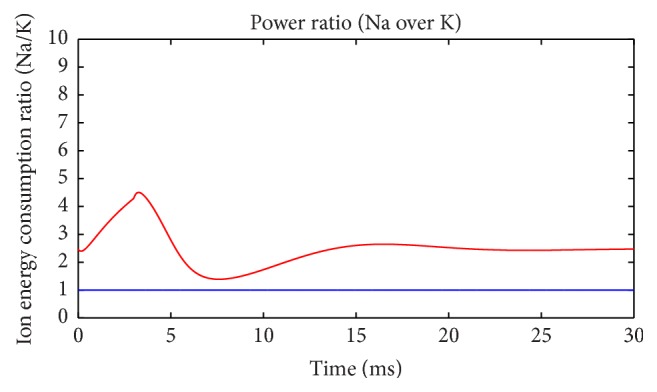
Power ratio of Na^+^ and K^+^ channels (subthreshold).

**Figure 15:**
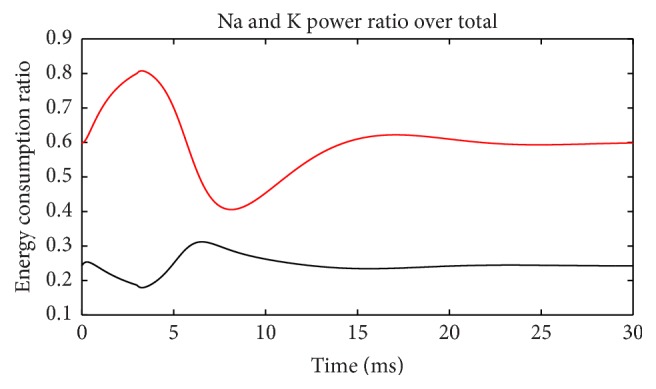
Power ratios of Na^+^ and K^+^ over total (subthreshold).

**Figure 16:**
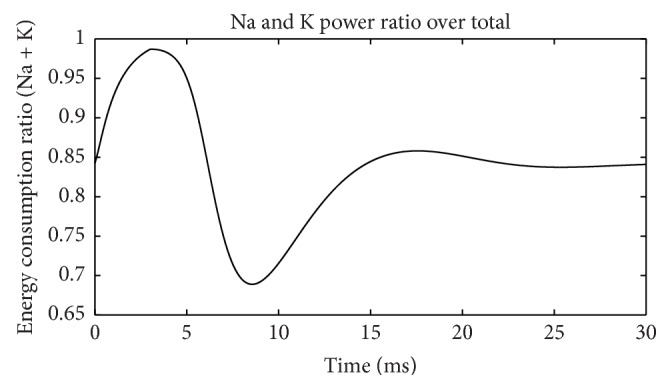
Sum of Na^+^ and K^+^ power over total power (suprathreshold).

**Figure 17:**
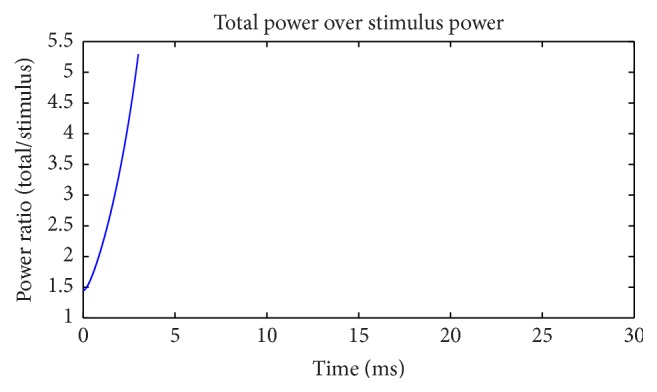
Ratio of total power and stimulus power (subthreshold).

Figure 13 shows the electric power consumption of a neuron during subthreshold activity. Meanings of colors are same as Figure 5. The differences are the fact that Na^+^ channel power is higher than K^+^ all the time and occupies most of the total power. The strengths of stimulus and leakage currents are the same order of magnitude as ion currents. The synchronicity of power *τ*(*E*) is 0.96, and phase difference Ψ(*E*) is 16.26°, suggesting that changing trends of these two ion powers are similar. Ignoring current directions, we can see that at firing state current synchronicity is higher than power (*τ*(*I*) = −0.987, *τ*(*E*) = 0.782), but, at subthreshold, it is just the opposite (*τ*(*I*) = −0.90, *τ*(*E*) = 0.96). The differences between current and power synchronicities are greater in the firing state than at subthreshold. The peak value of Na^+^ power is four times of K^+^, which is only 66% during action potential, suggesting further that Na^+^ channel uses most of the electric energy during subthreshold activity.  Figure 14 shows the power ratio of Na^+^ over K^+^, which is above the equality blue line all the time comparing with Figure 6 with four intersections. This proves again that the Na^+^ channel dominates the energy usage at subthreshold. However, Figure 11 suggests that K^+^ consumes less energy, and K^+^ currents are stronger than Na^+^ all the time. It is another interesting feature of the subthreshold activity. A strong current consumes less energy; this may be the effect of Nernst potential and voltage-gated channel. Power percentages of two channels are illustrated in Figure 15. Na^+^ power percentage is higher than 40% all the time while that of K^+^ is lower than 35%. It is clearly different from Figure 7. The sum of power percentages of Na^+^ and K^+^ is shown in Figure 16; the power stays above 70%. The rapid drop in Figure 8 never occurs. “Blasting fuse” effect of stimulus current does not occur either (Figure 17). Over time, the ratio of total power over stimulus power increases, suggesting that the more stimulus energy is provided to a neuron, the more electric power can be stimulated. However, the maximum of the ratio is <6, much less than the value of several hundred of thousand in Figure 9.

By the ion-counting method which integrates Na^+^ current we can get to 48.1 nC (per cm^2^) of positive electric charge flow into the cell membrane in the form of Na^+^ during subthreshold activity. These are 3.0 × 10^11^ of Na^+^ ions; then the number of moles of ATP is 1.66 × 10^−13^ mol. Finally, the energy supply to a neuron by ATP is 8.31 × 10^−9^ J. Meanwhile, integrating the total power with respect to time we will get the consumed electric power, which is 8.75 × 10^−9^ J. This is more energy than the ATP supplied. The energy efficiency is 105.3%. This is an anomaly during subthreshold activity that energy consumption is larger than energy supply. It can be explained that after stimulus current arrived and membrane potential fluctuate subthreshold, ion channels overdraft energy supply by ATP in advance during limited time for electric activity, which causes metabolic energy debt. Afterward, ATP should be consumed to repay the energy debt even during resting potential. However, a neuron receives stimulus randomly at any moment, so the energy debt remains. This may reveal the energy flow direction supplied to Na^+^/K^+^ pump by ATP on the ion and energy metabolism level. Part of this energy is used for repaying the energy debt, which is consumed in advance. This means a neuron consumes more energy than we anticipated during subthreshold activity.

### 3.3. Comprehensive Analysis

**Figure 18:**
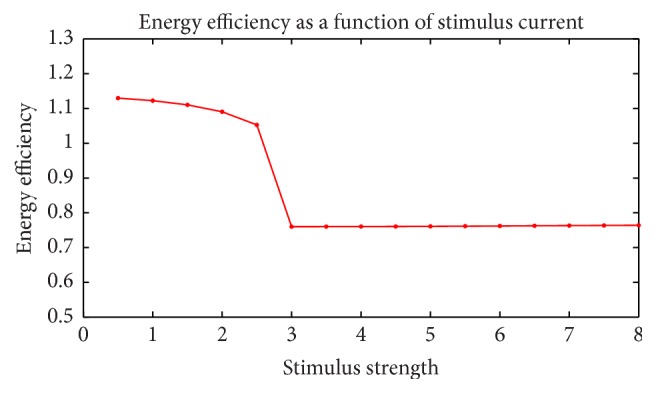
Energy efficiency as function of stimulus current strength.

**Figure 19:**
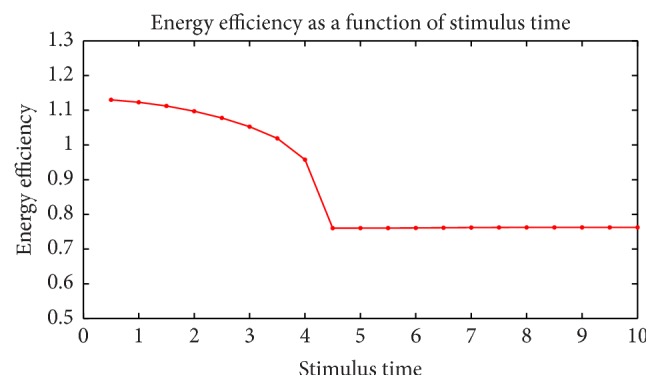
Energy efficiency as function of stimulus current duration.

Furthermore, we adjusted the strength and duration of the stimulus currents detect the change in energy efficiency.  Figure 18 shows the change in energy efficiency as a stimulus strength grows, while duration stays in 3 ms. It is the same in Figure 19, but the stimulus current keeps in 2.5 *μ*A and duration grows. These two figures have the following common features: (1) the trends are similar. Energy efficiency suddenly drops in the middle of the figures. This is the sign of switch from subthreshold to suprathreshold activity. (2) The figures decrease before the sudden drop and barely change after that. This is because membrane potential, ion currents vary a lot subthreshold while the action potential is identical. (3) Energy efficiencies are all above 100% subthreshold and remain approximately 76% suprathreshold.

## 4. Discussion

Based on the H-H model, we have studied currents and energy features of a neuron during different activity states with different stimulus. Regarding the neuron as a system with energy exchange with the environment, we distinguished, defined, and calculated the energy supply and energy consumption of a neuron. Then, the synchronicities of ion currents and ion channel power were defined. Furthermore, we studied the features and differences of ion currents and ion channel power at different neural activity states and found the following conclusions: All the ion migration across the membrane serves to neutralize the external stimulus charge injected into the cell during both sub- and suprathreshold activities. During suprathreshold activity, the strength relationship of Na^+^ and K^+^ currents fluctuates a great deal, while during subthreshold activity Na^+^ current is weaker than K^+^ all the time. The power relationship of these two channels fluctuates the suprathreshold and peak value of Na^+^ as 66% of K^+^. With subthreshold activity, Na^+^ power is stronger than K^+^ the entire time and the peak value is four times that of K^+^. Current synchronicity of these two ion channels is higher in suprathreshold than subthreshold activity, while power synchronicity is just the opposite. These two channels occupy most of the electric energy in both cases, but power ratio of Na^+^ is always larger than K^+^ at subthreshold state. Stimulus current shows a “blasting fuse” effect at both states, but the power ratio is much larger in suprathreshold case than the other. It is a very important feature that energy efficiency in subthreshold activity is close to or even more than 100%, which suggests that ion channels usually consume more energy than we thought and get into energy debt. Meanwhile, energy efficiency during an action potential is keeping around 76%. This essential difference could be regarded as the criterion of switch between subthreshold and suprathreshold activity; it can be observed from the energy efficiency figures that the efficiencies are divided into two groups automatically; the switch between two states can be easily found. In subthreshold activity, the more energy is injected by stimulus current, the lower energy efficiency is. However, the efficiency at firing state is stable.

The calculation suggested that the energy efficiency during an action potential is about 76%. This is consistent with published research by Attwell and Laughlin [26]. Their research revealed that 75% of energy expenditure in grey matter is devoted to signaling. Since action potential is the basis for signaling, our result perfectly matches their data. Research by Partadiredja et al. shows that a typical diameter of an axon is 10^−4^ mm [30]. Axon length is approximately 1 mm. Thus, the surface of an axon is about 3.14 × 10^−6^ cm^2^. Our calculation shows that 4.94 × 10^−12^ mol of ATP is consumed per cm^2^ during an AP (action potential). So the total number ATP molecules cost to transmit an AP along the axon is about 9 × 10^6^. This is close to the estimation made by Laughlin et al. that the metabolic cost for sensory information in insect retinas is 7 × 10^6^ ATP molecules per bit [25].

The total energy cost of one pump cycle, which pumps 3 Na^+^ ions out of the cell and two potassium ions in, can be estimated to be 0.37 eV [31]. During an AP, based on our calculation, in order to pump out the 8.918 × 10^12^ of Na^+^ ions which flow into the membrane, 2.468 × 10^−7^ J energy is consumed. It is easy to get that every 3 Na^+^ consumed 0.519 eV energy. The results are perfectly supported by published researches, both theoretical and experimental.

The amount of free energy released by per mole of ATP may vary in different situations, and this parameter value could perturb the calculation of energy efficiency. However, the distinct contrast of efficiency between the two states is certain. Neural subthreshold activity could overdraft the metabolic energy released by ATP; there may be some other unknown energy processes, except in ion channel consumption. How these unknown processes influence subthreshold energy debt should be further studied.

Neural activity and neural plasticity are the keys to solving the coding and decoding problems of neural information. Unfortunately, research techniques such as phase coding, frequency coding, and group coding have different kind of limitations and difficulties; there is still no complete theory for neural coding and decoding to direct the research of global brain activities. Under this circumstance, energy coding may be an alternative. Neuron and synaptic connection are the basic elements of neural system. So the energy properties of both neuron and synapsis should be studied. And only if the neural activity is thoroughly investigated, then the energy of synapsis and neural plasticity can be studied.

In this paper, we carefully revealed the energy properties of neural activity. It is the foundation of the further study of energy of synapsis and neural network. Neural energy and neural plasticity are closely related.

**Figure 20:**
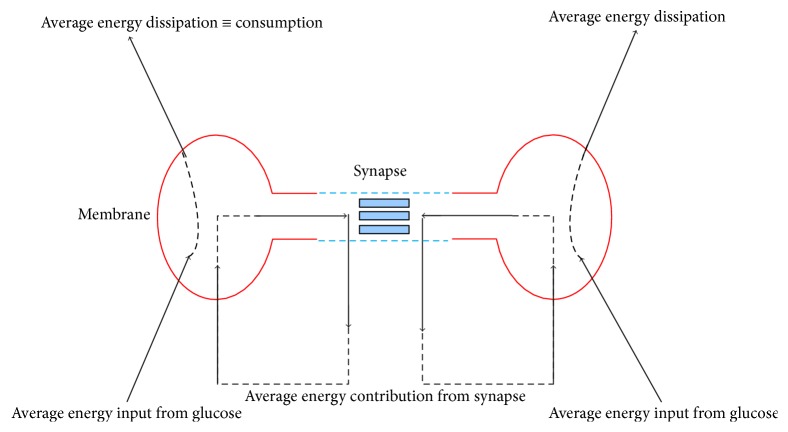
Global energy flow of neurons and synapse.

First, the synapse contributes energy to neural activity. It could be represented by the stimulus current in the model. Research has shown that some production of energy at the synaptic site is necessary for the neuron to keep its signaling [32]. There is biological evidence that links the generation of metabolic energy to the inflow of glucose through the membrane to produce ATP. Both facts could be reconciled assuming that the electrical energy produced at the synaptic site is conveniently transformed and reabsorbed by the neuron through its membrane for the generation of new spikes. A schema of the global energy flow of both neurons and synapse is provided in Figure 20 [32]. Energy of the synapse contributes significantly to the signaling and coding. As a result, neural plasticity links the neural energy to information signaling and transmission.

Second, synapse strength could be changed by neural energy. Neural plasticity is the basic mechanism of cognitive function of brain. The role of neural plasticity in energy coding method and how the neural energy affects synapse connections should be studied. In another work, we have proposed a synapse model to describe the strength change of synapse connection in energy level [33].

(12) kdωjidt=∫0∞PiτHτPjt−τ+Pit−τH−τPjτdτ.

This is a Hebbian learning rule in energy form. *P*_*i*_(*t*) and *P*_*j*_(*t*) are the power of neurons *i* and *j* at moment *t*; *k* is a constant and *H*(*t*) is a time window. It has been proven to be effective towards the construction of a neural network in energy form to solve certain cognitive problems [33].

Finally, to reveal the relationship of neural energy and neural plasticity is an important topic. The functional connection of brain area is defined by the simultaneous brain activity, which is observed by fMRI experimentally. And fMRI is based on the measurement of BOLD signal, which is essentially a reflection of neural energy. Thus, the theoretical research of neural energy benefits the study of synaptic connection of neurons.

To conclude, neural energy is closely related to neural plasticity. Synapse contributes energy to neural activity, and the activity energy of pre- and postsynaptic neurons changes the connection strength. Furthermore, a better understanding of neural energy will potentially support the research of functional brain connections.

Energy coding is a new coding method proposed from the perspective of global brain activity. The most important property of this method is linear additivity [19]. Almost every known process in nature can be seen as a certain kind of energy transformation or transmission. So can neural activity. However, countless neural processes are consuming energy. The linear additivity of the energy coding method may provide a possibility to obtain energy properties of global brain activity. Energy supply and consumption of a single neuron have been considered in this research. Combined with a proper synapsis energy consumption, more complex neural network energy properties can be studied. Under certain circumstances, the neural network energy should exhibit emerging properties, which contain more information about the neural system in the active mode and in neural coding. To conclude, energy method has a great potential, which could bring new surprises to the research of neural information coding and decoding.
